# Effects of Recombinant Human Growth Hormone Treatment, Depending on the Therapy Start in Different Nutritional Phases in Paediatric Patients with Prader–Willi Syndrome: A Polish Multicentre Study

**DOI:** 10.3390/jcm10143176

**Published:** 2021-07-19

**Authors:** Agnieszka Lecka-Ambroziak, Marta Wysocka-Mincewicz, Katarzyna Doleżal-Ołtarzewska, Agata Zygmunt-Górska, Anna Wędrychowicz, Teresa Żak, Anna Noczyńska, Dorota Birkholz-Walerzak, Renata Stawerska, Maciej Hilczer, Monika Obara-Moszyńska, Barbara Rabska-Pietrzak, Elżbieta Gołębiowska, Adam Dudek, Elżbieta Petriczko, Mieczysław Szalecki, on behalf of the Polish Coordination Group for rhGH Treatment

**Affiliations:** 1 Department of Endocrinology and Diabetology, The Children’s Memorial Health Institute, 04-730 Warsaw, Poland; m.wysocka@ipczd.pl (M.W.-M.); m.szalecki@ipczd.pl (M.S.); 2 Department of Paediatric and Adolescent Endocrinology, University Children’s Hospital, Jagiellonian University, 30-663 Krakow, Poland; drkate@tlen.pl (K.D.-O.); endodim@cm-uj.krakow.pl (A.Z.-G.); 3 Department of Endocrinology and Diabetology of Children and Adolescents, Wroclaw Medical University, 50-368 Wroclaw, Poland; kep@usk.wroc.pl (T.Ż.); anna.noczynska@umed.wroc.pl (A.N.); 4 Department of Paediatrics, Diabetology and Endocrinology, Medical University of Gdansk, 80-952 Gdansk, Poland; debirkhol@wp.pl; 5 Department of Endocrinology and Metabolic Diseases, Polish Mother’s Memorial Hospital-Research Institute, 93-338 Lodz, Poland; renata.stawerska@umed.lodz.pl (R.S.); maciej.hilczer@umed.lodz.pl (M.H.); 6 Department of Paediatric Endocrinology and Rheumatology, Institute of Paediatrics, Poznan University of Medical Sciences, 60-572 Poznan, Poland; m.moszynska@ump.edu.pl (M.O.-M.); b.rabska@ump.edu.pl (B.R.-P.); 7 II Clinic of Paediatrics, Endocrinology and Paediatric Diabetology, Clinical Regional Hospital No 2, 35-301 Rzeszow, Poland; sekretariat@szpital2.rzeszow.pl (E.G.); dudek.ad@wp.pl (A.D.); 8 Department of Paediatrics, Endocrinology, Diabetology, Metabolic Disorders and Cardiology of Developmental Age, Pomeranian Medical University, 71-242 Szczecin, Poland; elzbieta.petriczko@pum.edu.pl; 9 Collegium Medicum, Jan Kochanowski University, 25-317 Kielce, Poland

**Keywords:** recombinant human growth hormone, insulin-like growth factor 1, Prader–Willi syndrome, growth hormone deficiency

## Abstract

Recombinant human growth hormone (rhGH) treatment is an established management in patients with Prader–Willi syndrome (PWS), with growth promotion and improvement in body composition and possibly the metabolic state. We compared anthropometric characteristics, insulin-like growth factor 1 (IGF1) levels, metabolic parameters and the bone age/chronological age index (BA/CA) in 147 children with PWS, divided according to age of rhGH start into four groups, corresponding to nutritional phases in PWS. We analysed four time points: baseline, rhGH1 (1.21 ± 0.81 years), rhGH2 (3.77 ± 2.17 years) and rhGH3 (6.50 ± 2.92 years). There were no major differences regarding height SDS between the groups, with a higher growth velocity (GV) (*p* = 0.00) and lower body mass index (BMI) SDS (*p* < 0.05) between the first and older groups during almost the whole follow-up. IGF1 SDS values were lower in group 1 vs. other groups at rhGH1 and vs. groups 2 and 3 at rhGH2 (*p* < 0.05). Glucose metabolism parameters were favourable in groups 1 and 2, and the lipid profile was comparable in all groups. BA/CA was similar between the older groups. rhGH therapy was most effective in the youngest patients, before the nutritional phase of increased appetite. We did not observe worsening of metabolic parameters or BA/CA advancement in older patients during a comparable time of rhGH therapy.

## 1. Introduction

Recombinant human growth hormone (rhGH) treatment is a well-established proceeding in many indications in paediatric endocrinology. It is important in the management of not only patients with growth hormone deficiency (GHD) but also patients with with genetic disorders, such as Turner syndrome, Noonan syndrome or Prader–Willi syndrome (PWS) [1,2]. The treatment for patients with PWS is beneficial from different aspects of rhGH mechanisms of action. Growth promotion is only one of them, and it seems that a positive influence on the metabolic state, improvement in body composition and muscle strength and, therefore, improvement in psychomotor development play the most important role in this particular indication in the paediatric population [3,4,5,6,7]. Moreover, there are reports regarding a possible improvement in the health-related quality of life (HRQoL) in patients with PWS treated with rhGH, as well as a probable positive influence of rhGH therapy on cognition in the youngest children with PWS [8,9,10,11,12,13].

PWS is a rare genetic imprinting disease with hypothesised primary hypothalamic disorder caused by a lack of paternally inherited genes on chromosome 15q11-q13. The estimated prevalence is 1 in 15,000 to 1 in 30,000, with different categories of molecular diagnosis: paternal deletion (DEL 15, 65–75%), maternal uniparental disomy (UPD 15, 20–30%) or an imprinting centre defect (ID, estimated for 1–3%) [14,15,16,17,18,19]. The imprinted genes on chromosome 15q11-q13 include small nuclear ribonucleoprotein polypeptide N upstream reading frame–SNRPN (*SNURF-SNRPN*), makorin RING-finger protein 3 (*MKRN3*), MAGE family member L2 (*MAGEL2*), Necdin protein (*NDN*), chromosome 15 open reading frame 2 (*C15orf2*) and more than 70 C/D box snoRNA genes (*SNORDs*). Researchers are still trying to find an exact contribution of these genes to clinical manifestation in patients with PWS [20,21,22,23,24]. The main clinical features of PWS consist of different effects of hypothalamic disorders: a disturbed GH/IGF1 axis results in short stature and hypogonadotropic hypogonadism, with cryptorchidism in male newborns, and in delayed and incomplete puberty. A lack of feeling satiety results in obesity, developing from early childhood and leading, if untreated, to morbid obesity [14,15,16,17,18,19,25,26,27,28]. Other typical characteristics in children with PWS, apart from dysmorphic features, are marked hypotonia, global psychomotor delay, with moderate-to-severe difficulties in feeding in the first months of life, and usually cognitive and behaviour dysfunction in later childhood [14,15,16,17,18,19]. Animal studies have shown a possible link of the above-mentioned genes with different PWS features: *SNRPN,* involved in mRNA splicing in the central neurons, with hypotonia and impaired feeding, and other genes, expressed in the hypothalamus: *MAGEL2* with reduced motor activity, postnatal growth retardation and increase in adiposity and delayed puberty; *NDN* with reduced gonadal function; and *SNORD116* with hypotonia and a failure to thrive, followed by moderate hyperphagia [22].

However, recent studies show that the clinical picture of PWS is far more complicated than that previously described. Hypogonadism seems to be of both central and gonadal origin [29,30,31]. Moreover, a phase of premature adrenarche (PA) is frequently present in children with PWS [32]. What is of great importance is that we are now more aware that the natural history of PWS consists of different age-specific nutritional phases. In our research, we followed this division of nutritional characteristics in patients with PWS described by Miller et al. in detail in 2011 that was also predicted in earlier studies [3,33,34]. According to their findings, we can distinguish the following phases in children and adolescents (median age): 1a, until 9 months of life, with feeding difficulties and decreased appetite; 1b, until 25 months, characterised by improved feeding and appetite; 2a, until 4.5 years, with weight increase but yet without increased appetite; and 2b, until 8 years, when there is a beginning of increased appetite and calories intake. Above the age of 8 years, at phase 3, that lasts until reaching adulthood, patients show insatiable appetite. There are no differences reported in the median age of completion of the above phases in different types of molecular diagnosis, DEL 15 vs. UPD 15. The commencement of rhGH treatment in infants allows accelerating the pace of phase 1a and entering phase 1b earlier. It seems to be beneficial for improvement of feeding difficulties and marked hypotonia in the early age [34].

The exact hormonal or biochemical background for this sequence of events is still not fully documented. It seems to be an interesting field for further research to explain how hypothalamic dysfunction develops over time and whether the impaired growth hormone/insulin-like growth factor 1 (GH/IGF1) axis contributes to these changes in the nutritional phenotype. These aspects could bring light to a better understanding of not only the background of PWS but also other hypothalamic disorders with sometimes challenging management, such as hypothalamic tumours.

Moreover, another important question is whether and how we can modify the nutritional phases of patients with PWS with a clinical approach: rhGH treatment together with multidisciplinary care. Again, the possible positive effects of the therapy in modifying the natural history of PWS may be useful in the management of other endocrine conditions.

To try to answer this question, we analysed the data of a large cohort of children with PWS treated with rhGH for a long period in relation to therapy commencement in the different nutritional phases of the disease. We compared the treatment effects in terms of anthropometric characteristic improvement, as well as the influence on IGF1 levels, metabolic parameters and bone maturity advancement.

## 2. Material and Methods

Medical records and questionnaires filled in by clinicians regarding the data of 147 patients with PWS (69 girls (46.94%), 78 boys (53.06%)) from 12 paediatric endocrine centres in Poland, treated with rhGH in the years 2002-2016, were retrospectively analysed. The rhGH treatment was accepted by the Polish Coordination Group for rhGH Treatment. We recently published the first part of the data analysis regarding perinatal characteristics, anthropometric data and biochemical data before commencement of rhGH treatment in relation to the genetic subtype and the age of genetic diagnosis [35].

In this study, we focused on the effects of rhGH therapy in relation to the age of rhGH start. We compared four groups of patients, according to the above-described age-dependent nutritional phases: group 1, age ≤2 years; group 2, age >2 and ≤4.5 years; group 3, age >4.5 and ≤8 years; and group 4, age >8 years. Groups 1 and 2 correspond to the nutritional phases in children with PWS when increased appetite is still usually not present (phases 1a, 1b, and 2a). Groups 3 and 4 are in concordance with the phases of increased appetite (phases 2b and 3), and especially in group 4, hyperphagic behaviour may be a dominant characteristic.

The percentage of the exact molecular type of diagnosis was as follows: DEL 15, *n* = 81 (55.10%); UPD 15, *n* = 10 (6.80%); and excluded DEL 15 (UPD/ID), *n* = 30 (20.41%). In 7 patients of the last subgroup (4.76% of the whole cohort), DEL 15 was excluded, but further molecular studies were not documented. The abnormality in the methylation pattern of *SNRPN*, diagnostic for PWS, was confirmed in 36 patients (24.49%), with the exact type of genetic diagnosis pending; in 1 patient the genetic report was not included in the available medical files. In previous research, cited above, regarding the correlation of genotype and perinatal period, time of diagnosis and data before rhGH start in our group of patients, we discussed the lack of specific molecular diagnosis in part of the cohort, as well as in other published data of large groups of patients with PWS, and the need for improvement in this field [35].

We compared the available data at four time points of follow-up (mean time ± SDS): baseline, rhGH1 (1.21 ± 0.81 years of rhGH treatment), rhGH2 (3.77 ± 2.17 years of rhGH treatment) and rhGH3 (6.50 ± 2.92 years of rhGH treatment). We also looked in more detail at the therapy effects within each group separately.

The height and body mass index (BMI) were assessed according to the Polish growth and BMI standard charts, BMI SDS were calculated using the LMS method (method to obtain the SDS, LMS parameters: lambda for the skew, mu for the median and sigma for the generalised coefficient of variation). IGF1 and insulin levels were evaluated with a radioimmunoassay technique. Conversion of IGF1 results to one reference was necessary as different assays were used in different centres and within 15 years of follow-up. The oral glucose tolerance test (OGTT) was performed with a glucose dose of 1.75 g/kg (maximal dose 75 g). Additionally, insulin resistance was estimated with the homeostasis model assessment of insulin resistance (HOMA-IR). Lipid profile assessment included total cholesterol (TC), high-density lipoprotein cholesterol (HDL-C), low-density lipoprotein cholesterol (LDL-C) and triglycerides (TG). Bone age (BA) measurements were evaluated according to the Greulich and Pyle atlas, and the bone age/chronological age index (BA/CA) was calculated. The study was approved by the CMHI Bioethics Committee (7/KBE/2019, 20 March 2019).

## 3. Data Analysis

Statistica v13 (TIBCO Software Inc. (2017), Palo Alto, CA, USA and StatSoft Company, Tulsa, OK, USA) was used for statistical analyses. Results were expressed as mean values and standard deviation scores (SDS) and additionally as the median (minimal–maximal value). Data were checked for normality of distribution using the Shapiro–Wilk test, and data with skewness were log- or square-transformed to a normal distribution, if possible. Differences between groups were tested by the unpaired Student’s t-test or the Mann–Whitney U test, as appropriate. Differences between dependent variables were analysed by Student’s t-test for dependent variables or the Wilcoxon test, as appropriate. Variables from 4 time points (3 time points for growth velocity (GV) data) for separate groups were analysed using the Friedman test for dependent variables because of a lack of distribution normality or variance homogeneity of at least one variable in a group. A *p* level of <0.05 was considered as statistically significant.

## 4. Results

### 4.1. Molecular Type of Diagnosis and Patients’ Clinical Characteristics

**Figure 1:**
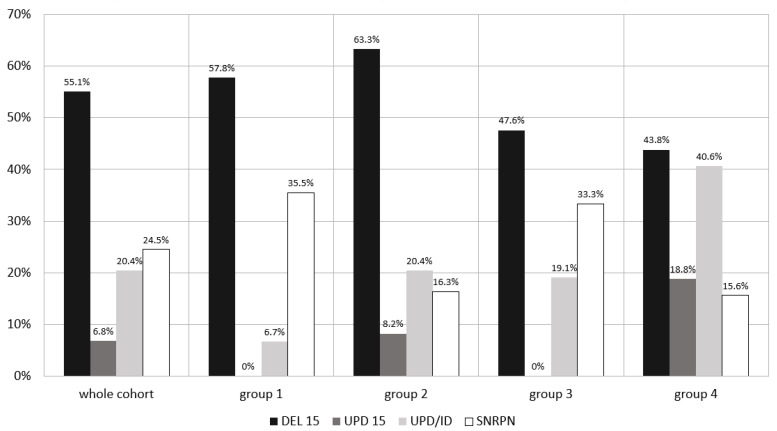
Frequency of molecular types of diagnosis in the whole study cohort and the separate groups (percentage). Group 1: age ≤ 2 years; group 2: age >2 and ≤4.5 years; group 3: age >4.5 and ≤8 years; and group 4: age >8 years of life. DEL 15—deletion of chromosome 15q11-13; UPD 15—uniparental disomy; ID—imprinting defect; SNRPN—abnormality in methylation pattern of *SNRPN*.

The details regarding the genetic diagnosis and clinical features of the patients are presented in Table 1 and, additionally, the percentage of different molecular types of diagnosis in Figure 1.

According to the type of genetic diagnosis, we observed that the highest number of UPD/ID was present in the eldest group of patients. Children in group 1 were diagnosed at the earliest age compared with all the other groups (*p* = 0.00).

### 4.2. Age of rhGH Start and rhGH Dosage

rhGH treatment was started at the mean age of 4.50 ± 3.73 years, median 3.03 (0.58–17.43), with a mean rhGH dose of 0.58 ± 0.16, median 0.56 (0.22–1.40) IU/kg/week, i.e., 0.028 ± 0.008, median 0.027 (0.011–0.067) mg/kg/day. A dose of 0.067 mg/kg/d is a higher limit of rhGH dosage in children with PWS recommended by the Polish Coordination Group for rhGH Treatment. There were no significant differences in rhGH doses between the four groups of patients. In 65 (46.43%) patients, the dose was changed with time, mainly decreased (groups 2–4) to 0.50 ± 0.17 IU/kg/week (0.024 ± 0.008 mg/kg/day) and changed further in 26 (20.64%) patients to 0.42 ± 0.19 IU/kg/week (0.02 ± 0.009 mg/kg/day). In four patients, rhGH treatment was continued with the lower, metabolic dose due to the end of the growth promotion period (rhGH therapy for adults with PWS has been available in Poland since 2016). In 13 (10.32%) patients, the dose had to be decreased due to high levels of IGF1 (>2 SDS). However, in the youngest group, with relatively lower IGF1 values, the mean rhGH dose was increased in 12 patients (27.27%) to 0.61 ± 0.15 IU/kg/week (0.029 ± 0.007 mg/kg/day), followed by a change in 4 patients (11.43%) to 0.67 ± 0.30 IU/kg/week (0.032 ± 0.01 mg/kg/day) during follow-up. The above percentage rates may not be fully accurate as some information regarding changing the dose lacked the exact date.

### 4.3. Effects of the Therapy in the Whole Cohort of Patients and Comparison between the Separate Groups

The effects of therapy at three time points were analysed in a consecutive number of patients (percentage): 140 (95), 126 (86) and 99 (67) (Table 2 and Table 3).

Not all the patients presented at every time point of rhGH therapy. Treatment was finished in 24 patients at the mean age of 13.28 ± 4.91 years, in 15 of them prematurely mainly due to a significant increase in obesity, despite the multidisciplinary care. One girl from group 2 died at the age of 5.2 years during a second surgery for congenital heart disease—hypoplastic right ventricle after 2.8 years of rhGH treatment.

It is necessary to note that our study analysis started before rhGH therapy was widely available for children with PWS in Poland (2006), when patients were treated with the families’ own resources, and ended at the time when rhGH therapy was implemented for adult patients with PWS in Poland (2016).

We did not find differences regarding height SDS before and during the treatment between the first and other groups. The only significant difference was present at rhGH2 for group 4 vs. group 3 and at rhGH3 for group 4 vs. group 2, with lower height SDS in the eldest group (*p* = 0.028 and *p* = 0.037, respectively).

Analysis of GV revealed an expected higher GV during almost the whole rhGH therapy between the first group compare with the older groups of patients (p = 0.00, except for group 1 vs. 2 at rhGH2). When we compared group 2 vs. 3, a similar difference was present at rhGH2 (*p* = 0.01), with only a tendency at rhGH3 (*p* = 0.066). Comparison of group 2 vs. 4 showed a higher GV during the whole therapy in group 2 (at rhGH1: *p* = 0.01; at rhGH2 and rhGH3: *p* = 0.00). Comparing the eldest groups, 3 and 4, we again found a higher GV in group 3 at rhGH2 (*p* = 0.01), with only a tendency towards a higher GV at rhGH3 (*p* = 0.059).

A significant difference in BMI SDS was observed before and throughout the whole follow-up between group 1 and all the other groups, with lower BMI SDS in the first group (*p* < 0.05). Interestingly, there was no difference in BMI SDS between the older groups, with only one exception (lower BMI SDS in group 2 vs. 4 at baseline, *p* = 0.00).

At baseline, IGF1 SDS were significantly higher in the first group only when compared with the eldest group. With time of rhGH therapy, this difference was reversed and IGF1 SDS were lower in group 1 vs. all other groups at rhGH1 and vs. groups 2 and 3 at rhGH2. Similar results at baseline were present for group 2 vs. 4, but higher IGF1 SDS in group 2 were also documented at rhGH 2 and rhGH 3. IGF1 SDS were also higher in group 3 vs. 4 at rhGH2.

### 4.4. Effects of the Therapy—Comparison of Four Time Points of Follow-Up

We also looked in more detail at the data at every time point during rhGH treatment within the whole study cohort (Table 2) as well as within the separate groups.

Most importantly, height SDS were expectedly significantly higher at rhGH1 compared with baseline, at rhGH2 vs. rhGH1 and at rhGH3 vs. rhGH2 within the whole cohort and within most of the four groups of patients analysed separately. However, this difference diminished with the age of the patients and is still significant within groups 1-3 at rhGH2 vs. rhGH1 but is not present in group 4. In both eldest groups, 3 and 4, height SDS did not reveal a significant difference at rhGH3 vs. rhGH2. GV values within the whole cohort and within the groups significantly decreased during rhGH therapy (rhGH2 vs. rhGH1, rhGH3 vs. rhGH2), except at rhGH3 vs. rhGH2 in group 1.

BMI SDS showed a steady significant rise within the whole cohort since rhGH1 (1.21 ± 0.81 years of rhGH treatment). However, within the separate groups, we found only the following significant differences: group 4—BMI SDS at rhGH1 were lower in comparison to basal BMI SDS (*p* = 0.01). At rhGH2 vs. rhGH1 and rhGH3 vs. rhGH2, again a difference was observed in group 4 but this time with higher BMI SDS at rhGH2 and rhGH3 (*p* = 0.04 and *p* = 0.004, respectively), and in group 2 at rhGH3 vs. rhGH2 as well, with higher BMI SDS at rhGH3 (*p* = 0.03).

**Figure 2:**
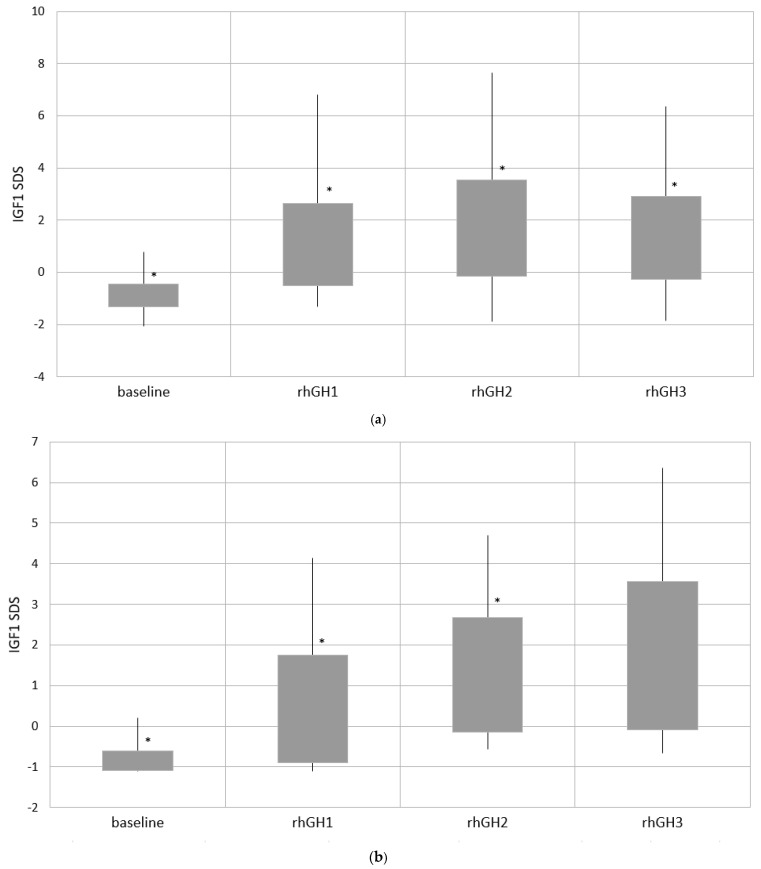

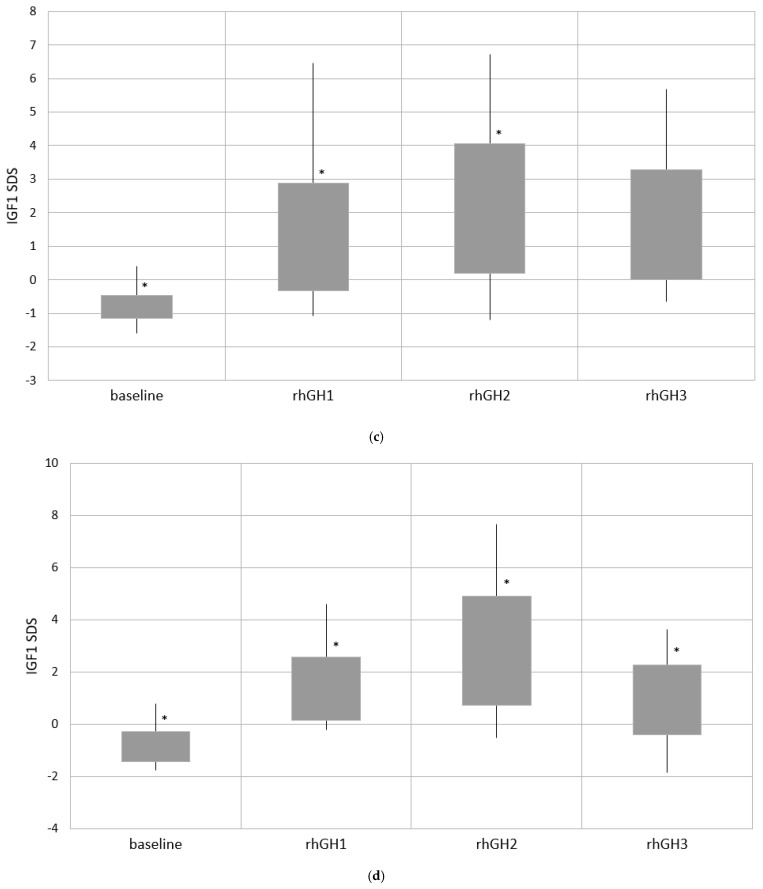

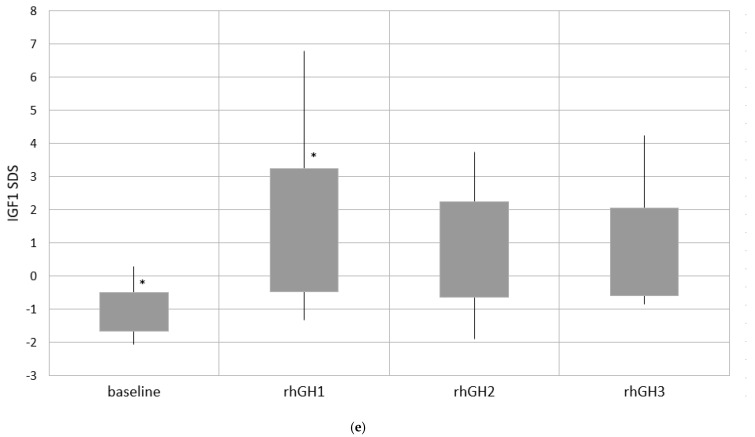
Insulin-like growth factor 1 (IGF1) SDS at baseline and during recombinant human growth hormone (rhGH) treatment. The bottom of the box indicates -1 SDS; the top of the box, +1 SDS; and the vertical lines, minimal and maximal values. * *p* < 0.05 for the mean values: rhGH1 vs. baseline, rhGH2 vs. rhGH1 and rhGH3 vs. rhGH2. (**a**) Whole cohort and (**b**) group 1: age ≤2 years of life; (**c**) group 2: age >2 and ≤4.5 years of life; (**d**) group 3: age >4.5 and ≤8 years of life; and (**e**) group 4: age >8 years of life.

Regarding IGF1 SDS, there was a strong significant rise within the whole study cohort and within separate groups until rhGH 2 (except rhGH2 vs. rhGH1 in group 4). However, when comparing IGF1 SDS at rhGH3 vs. rhGH2, this difference was not present, and furthermore, at rhGH3, IGF1 SDS were significantly lower within the whole group and group 3. Importantly, in the whole cohort, IGF1 values exceeded 2 SDS in 31 (22.14%) patients at rhGH1, 51 (42.86%) at rhGH2 and 26 (28.26%) at rhGH3. The differences in IGF1 SDS within the whole cohort and separate groups at four time-points of follow-up are presented in Figure 2.

When analysis of variance was performed using the Friedman test for dependent variables from four time points for separate groups, we confirmed a statistically significant difference regarding BMI SDS in groups 2 and 4 (*p* = 0.00). Moreover, this difference also occurred in group 3 (*p* = 0.04). All other characteristics (height SDS, GV (three time points) and IGF1 SDS) showed a statistically significant difference in each group during follow-up (*p* = 0.00).

### 4.5. Metabolic Parameters Evaluation

**Figure 3:**
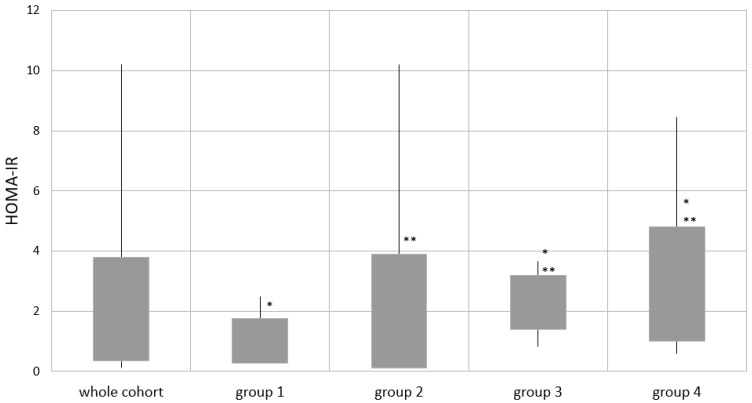
HOMA-IR in the whole study cohort and separate groups. the bottom of the box indicates -1 SDS; the top of the box, +1 SDS; and the vertical lines, minimal and maximal values. HOMA-IR—Homeostasis Model Assessment of Insulin Resistance; group 1: age ≤2 years; group 2: age >2 and ≤4.5 years; group 3: age >4.5 and ≤8 years; and group 4: age >8 years of life; *p* < 0.05 for the mean values: * group 1 vs. 3 and 1 vs. 4; ** group 2 vs. 3 and 2 vs. 4.

The mean values of OGTT results were within the normal range in the whole study cohort as well as in separate groups (Table 4). However, the mean HOMA-IR results exceeded 2 in the whole study population and in the eldest groups, 3 and 4 (Figure 3).

Group 1 had significantly lower glucose and insulin levels in the OGTT (fasting glucose and both fasting and 120 min insulin), with lower HOMA-IR values, in comparison to groups 3 and 4. However, patients from group 1 had been assessed as younger children and after a shorter period of rhGH therapy. Within the older groups, 2-4, with a comparable time of rhGH treatment, fasting glucose and insulin levels with HOMA-IR were lower in the younger group 2 (group 2 vs. 3 and group 2 vs. 4, except for fasting insulin in group 2 vs. 3). The differences were not observed when comparing the eldest groups: 3 vs. 4.

We observed impaired fasting glucose in 2 and impaired glucose tolerance in 10 patients (together 13.19% of available results). Insulin resistance was recognised in 25 patients (27.47%) on the basis of increased insulin levels in the OGTT, defined as a fasting insulin level above 15 uIU/mL and/or an insulin level at 120 min of above 75 uIU/mL, and in 36 patients (39.56%) with HOMA-IR results >2. Diabetes mellitus type 2 (DM2) was present in one girl from group 2, she was treated with rhGH for 11.2 years and the therapy was discontinued due to DM2. We did not find any difference between the groups regarding the lipid profile. Lipid disorders, mainly hypercholesterolaemia, and less often hypertriglicerydaemia, were present in 31 patients (34.07%).

### 4.6. Bone Maturation Assessment

Regarding BA/CA analysis, there was an expected difference between the first group vs. all the other groups, with decreased BA/CA in the youngest patients after the shortest time of rhGH treatment. We did not observe differences in BA/CA (results close to 1) between the older groups, 2–4, during a comparable time of rhGH treatment (Table 5).

### 4.7. Adrenarche and Central Puberty Evaluation

PA was observed in 22 patients (14.97%) of the whole study cohort, with the highest frequency in group 3–7 patients (33.33%) (Table 1).

Spontaneous central puberty started in some of the patients in groups 2–4. However, during the study, a significant part of the cohort was still at the age that did not allow us to finally confirm hypogonadism. Some of the patients started sex hormones (testosterone/oestrogen/oestrogen-progesterone (T/E/E-P)) therapy either to induce the puberty process or due to the incomplete course of central puberty (Table 1). Overall, the central puberty features were present in 17 patients (34.69%) in group 2, 13 (61.91%) in group 3 and 21 (65.63%) in group 4.

## 5. Discussion

The main results of our study show that although rhGH therapy is most effective in the youngest patients, before the nutritional phase of increased appetite, we can still observe stable BMI SDS maintenance and improvement in height SDS in the first period of the treatment, commenced in the early phase of increased appetite.

It has been shown in numerous studies that rhGH therapy has a beneficial role in management of children with PWS. The mechanism of rhGH action is multidirectional, which we can clearly observe in patients with PWS [4,5,6,7,36,37,38,39,40,41]. Here, we tried to look at the clinical settings of rhGH action in children with PWS, the disease with a background of multiple hypothalamic disorders, with a special insight into different nutritional phases reported in PWS.

Analysis of the genetic background of the study population confirmed our previous research conclusions that a UPD/ID genetic background seems to be recognised later in life and therefore these patients start rhGH therapy at an older age [35]. Moreover, as expected, the earliest genetic diagnosis leads to the earliest rhGH commencement. Within the study group, close to 25% of patients did not have the exact molecular diagnosis. This lack of a precise type of genetic diagnosis has also been observed in recently published retrospective studies [1,38,39,42]. However, we still need to remember that our study regarded a long period and treatment started several years ago when genetic analysis as well as rhGH treatment were not widely available in Poland.

In the literature, the estimated frequency of GHD in patients with PWS is reported as 40–100%; in a large French cohort (*n* = 142), GHD was present in 80% of patients [40,41,43]. However, GHD in adults seems to be less obvious, and the criteria for GHD are biochemically fulfilled in 8–38% of the studied cohorts [44]. The prevalence of GHD in patients with PWS is reported independently of the obesity presence [45]. Interesting data show that children with PWS seem to be more sensitive to rhGH treatment. In a study comparing rhGH therapy effects in children with PWS vs. patients with mainly idiopathic GHD, a higher GV during the first 6 months of treatment was found in patients with PWS, despite the significantly lower rhGH dose [45]. Another study showed markedly high acid-labile subunit (ALS) levels in rhGH-treated patients with PWS compared with controls [46]. Most of the serum IGF1 is present in complexes with IGF1 binding protein 3 (IGFBP-3) or IGFBP-5 and ALS. As ALS production is mainly regulated by GH, this finding again suggests that children with PWS are more sensitive to rhGH therapy. Moreover, ALS has been shown to be involved in carbohydrate and fat metabolism in animals, and it is hypothesised that higher levels of ALS may also help to improve the metabolic state in patients with PWS during rhGH treatment [46].

Analysing the anthropometric data, we found lower rhGH effectiveness in terms of height SDS and GV improvement in older children while being treated for a longer time. The observed decrease in the GV during follow-up is expected, as GV improvement is usually the highest in the first two years of rhGH treatment in all paediatric indications. In our study, only the youngest group, children who started the therapy below the age of 2 years, had a comparable GV at rhGH3 (4.3 years of rhGH therapy) vs. rhGH2 (2.9 years); therefore, the effect regarding improving the GV was sustained. These results are in concordance with a thesis that the younger the patients with PWS, the better the GV reached during rhGH therapy. However, it is also the age of one of the physiologic paediatric phases of a high GV. Moreover, in our study, the time of treatment was the shortest in these patients, and it could partially result in a difference in the GV and in better results at the last time point. Height SDS improved significantly throughout rhGH therapy in the study population, and this improvement was seen for the two youngest groups until rhGH3. Interestingly, with a few exceptions, we did not observe significant differences in height SDS during the therapy among the groups despite differences in the pubertal stage. However, we documented scoliosis, which is typical for patients with PWS, in 23% of the study group, with the highest frequency in the eldest patients. This could have had an additional important impact on the assessment of height SDS during follow-up.

BMI SDS in the research group support the hypothesis that the earliest rhGH start allows to fully maintain the excess weight gain in children with PWS. However, this result may be difficult to assess as the initial BMI SDS in the youngest group were lower than in the other groups. Moreover, during follow-up, patients in group 3, who entered the phase of increased appetite, also maintained similar BMI SDS. We observed a significant increase in BMI SDS mainly in the eldest group, along with the time of rhGH treatment (rhGH2 and rhGH3), despite the initial positive influence on BMI SDS (rhGH1). The presented results are in concordance with the hypothesis that early rhGH therapy commencement, before the nutritional phase of increased appetite, helps to maintain favourable BMI SDS in children with PWS. However, even in the early stage of increased appetite, the stabilisation of BMI SDS is observed. The results of a study regarding dietary energy intake during rhGH therapy, published in 2015, are worth mentioning [47]. The authors analysed a group of 47 children with PWS before and during the treatment and reported an increase in energy intake during two years of rhGH treatment but also an improvement in body composition and adiponectin levels, which may suggest a protective effect of rhGH.

Regarding monitoring the safety of rhGH treatment, we found the expected significant rise of IGF1 SDS until rhGH2 (mean time of follow-up: 3.8 years). Further observation showed either stabilisation or lowering of IGF1 at rhGH3 (6.4 years of therapy). The mean IGF1 SDS before the start of rhGH therapy were close to -1, and the maximal value did not exceed 0.8. It may result from the expected GHD or other subtle disorders of the GH/IGF1 axis in patients with PWS. Moreover, the youngest children, in the phase of feeding difficulties, may have lower IGF1 levels caused by undernourishment. During the treatment, although the mean IGF1 SDS were within the normal range, we observed a significant part of the results above 2 SDS, despite a relatively low rhGH dose. The highest values, with a higher percentage of IGF1 SDS above 2, reaching 43%, were presented at rhGH2 for the whole study cohort. In 10% of the patients, this led to a necessity of decreasing rhGH doses, which could result in lowering the IGF1 SDS at rhGH3, with lowering the frequency of high IGF1 SDS to 29%. The lowest IGF1 SDS during rhGH therapy were present in the youngest group, although this difference diminished at rhGH3. We have to consider the possible physiological rise in IGF1 SDS. However, due to hypothalamic–pituitary disturbances in GH/IGF1 as well as hypogonadotropic axes, this influence is not straightforward. The safety of higher IGF1 levels has been previously studied, and it is hypothesised that bioactive IGF1 values in patients with PWS stay within the norms during rhGH therapy [45,46]. In the study cited above, regarding rhGH treatment in children with PWS compared with patients with GHD, it has been shown that although IGF1 SDS exceeded the normal values in the PWS group, there was a lack of change in the IGF1-to-IGFBP3 molar ratio [45]. In another research, higher IGF1 bioactivity was present only in young rhGH-treated children with PWS compared with matched healthy controls, whereas this difference diminished in older patients [46]. It seems that both IGF1 and IGF1BP3 should be routinely monitored during follow-up for obtaining better insight into bioactive IGF1 levels. However, the recommendations for management of patients with PWS still include the necessity of maintaining IGF1 levels within the upper part of a normal range (maximum 2 SDS) for age and sex [4,48].

Miller et al. reported low IGF1 values in nutritional phase 1, with a significant rise in phase 2a, while on a stable rhGH dose of 0.029–0.037 mg/kg/d, suggesting a change in the rate of GH metabolism. The difference was not further seen in groups 2b and 3 [34]. We found higher IGF1 SDS as well in the older groups but not through the whole follow-up. A similar rhGH dose was used in our study population, i.e., 0.028 ± 0.008, median 0.027 (0.011–0.067) mg/kg/day. Although the approved rhGH dosage for patients with PWS is usually higher (1.0 mg/m^2^/d, which corresponds to 0.033–0.035 mg/kg/d), it is recommended to start therapy with a lower dose of 0.5 mg/m^2^/d [4,48]. The latest studies confirm the sustained effectiveness in terms of auxological response and safety of lower rhGH doses in infants with PWS (0.64 mg/m^2^/d) [49]. However, earlier rhGH dose–response studies have shown that a lower dose may not be beneficial for optimising and maintaining the improvement in body composition [3,7,38,50].

There is still an open issue of the influence of rhGH on metabolic homeostasis in children with PWS. Most of the studies confirm the observation that long-term rhGH treatment dose not adversely influence glucose metabolism [51]. The fasting insulin values in a study by Miller et al. were higher in phase 2b but without differences in fasting glucose levels [34]. In our analysis, the mean values of glucose and insulin in the OGTT, as well as the lipid profile, were within the normal range. Overall, the metabolic state assessment showed that younger children present better results of glucose homeostasis, which may be the result of the younger age itself. This difference diminishes with the age of the patients but does not show significant worsening in the eldest groups. Although the overall mean values of metabolic parameters are still within the normal range during rhGH treatment, we may observe metabolic disorders in a significant proportion of children with PWS. The frequency of insulin resistance reached 40% and dyslipidaemia 34% of the available tests. However, there are many factors that may influence these results, such as BMI rise during therapy and, again, the possible physiological phase of insulin resistance. In this study, we did not look in detail at the possible influence of central puberty start or implementation of sex steroids on glucose metabolism. However, a lack of difference in insulin resistance results between the two eldest groups suggests that this influence is not the most important factor. It is consistent with the observation made by Miller et al. in their cohort of children in different nutritional phases [34].

Interestingly, in patients with PWS, relative hypoinsulinaemia has been previously described. DM occurs mainly in adulthood, in patients with severe obesity, with an estimated prevalence of 10–25% [51,52,53]. In our observation, only one girl was diagnosed with DM2 during follow-up.

We did not observe significant BA/CA advancement during follow-up when comparing the older groups of patients with rhGH start at >2 years of life. Regarding the adrenarche period, PA was present in almost 15% of the whole cohort. However, in group 3, with the age typical for the signs of PA (>4.5 and ≤8 years), the PA frequency reached 33%, which is more consistent with previous studies [32,54,55]. We recently published a study showing that PA does not seem to influence the effectiveness and metabolic safety of rhGH therapy for children with PWS [32]. Lastly, there were no observations of neoplasm occurrence during the whole study.

Interestingly, recent findings show a probable positive influence on brain development via the GH/IGF1 axis and therefore on cognitive improvement when rhGH treatment is introduced very early in infancy, although this is still being discussed [10,11]. This is a field for further studies of the influence of rhGH on central nervous system development in infancy.

We acknowledge the limitations of our study that result mainly from the retrospective character of the research. Although the same questionnaires were filled in by clinicians from all the endocrine centres, no strict standardisation for the site documentation was present. Strengths of the study result from the high number of patients despite the rare character of the disease and the relatively long follow-up.

## 6. Conclusions

On the basis of the results of our study, we may conclude that rhGH therapy is most effective in terms of improving height SDS and maintaining both GV and BMI SDS in the youngest patients, before the nutritional phase of increased appetite. In the early phase of increased appetite, we can still observe maintaining stable BMI SDS and improving height SDS in the first years of rhGH treatment. This supports the recommendation for commencement of rhGH therapy early in children with PWS for the most beneficial effects regarding the nutritional phenotype.

We did not observe worsening of the metabolic state in the older patients. Moreover, there was no significant BA/CA advancement during follow-up in the older patients. However, a significant part of our study group presented with high IGF1 levels, and this should be further monitored.

## Figures and Tables

**Table 1:** Type of genetic diagnosis and clinical characteristics of patients with PWS, number of patients (percentage), mean value ± standard deviation scores (SDS) and median (minimal–maximal value) for the age of diagnosis.

PWS Group	Whole Cohort	Group 1	Group 2	Group 3	Group 4
Number of patients	*n* = 147	*n* = 45 (30.61)	*n* = 49 (33.33)	*n* = 21 (14.29)	*n* = 32 (21.77)
F/M	69/78 (46.94/53.06)	25/20 (55.56/44.44)	25/24 (51.02/49.98)	10/11 (47.62/52.38)	9/23 (28.13/71.87)
Age of diagnosis (years) ^a^	1.67 ± 2.39 0.53 (0.02–12.49)	0.29 ± 0.29 0.20 (0.04–1.22)	0.79 ± 0.83 0.44 (0.02–2.85)	2.98 ± 2.12 3.06 (0.15–7.31)	4.24 ± 3.40 3.54 (0.02–12.49)
DEL 15	81 (55.10)	26 (57.77)	31 (63.26)	10 (47.62)	14 (43.75)
UPD 15	10 (6.80)	none	4 (8.16)	none	6 (18.75)
UPD/ID	30 (20.41)	3 (6.67)	10 (20.41)	4 (19.05)	13 (40.62)
SNRPN	36 (24.49)	16 (35.56)	8 (16.33)	7 (33.33)	5 (15.63)
Cryptorchidism	70 (89.74)	19 (95.00)	23 (95.83)	8 (72.73)	20 (86.96)
PA	22 (14.97)	3 (6.67)	8 (16.33)	7 (33.33)	4 (12.50)
T/E/E-P	26 (25.49) ^b^	none	6 (12.25)	5 (23.81)	15 (46.88)
Secondary hypothyroidism	51 (34.69)	14 (31.11)	14 (28.57)	12 (57.14)	11 (34.38)
Scoliosis	34 (23.13)	3 (6.67)	15 (30.61)	4 (19.05)	12 (37.50)
Adenoid hypertrophy	28 (19.05)	2 (4.44)	12 (24.49)	6 (28.57)	8 (25.00)
Severe behaviour disorders	17 (11.57)	none	3 (6.12)	2 (9.52)	12 (37.50)
Epilepsy	12 (8.16)	2 (4.44)	3 (6.12)	3 (14.29)	4 (12.50)

PWS—Prader–Willi syndrome; n—number of patients; F—female; M—male; DEL 15—deletion of chromosome 15q11–13; UPD 15—uniparental disomy; ID—imprinting defect; SNRPN—abnormality in methylation pattern of *SNRPN*; T/E/E-P—testosterone/oestrogen/oestrogen–progesterone therapy; PA—premature adrenarche; ^a^ *p* < 0.05—age of diagnosis between all groups; ^b^ percentage of T/E/E-P therapy in groups 2–4.

**Table 2:** Data during rhGH treatment in the whole cohort of patients with PWS, expressed as the mean value ± standard deviation scores (SDS) and median (minimal–maximal value).

rhGH Therapy	Baseline	rhGH1	rhGH2	rhGH3
Number of patients (%)	*n* = 147	*n* = 140 (95)	*n* = 126 (86)	*n* = 99 (67)
Age of patients (years)	4.50 ± 3.73 3.03 (0.58–17.43)	5.73 ± 3.89 4.17 (0.89–19.96)	8.26 ± 4.27 7.12 (1.85–17.52)	11.06 ± 4.71 10.67 (2.72–20.67)
Time of rhGH therapy (years)	NA	1.21 ± 0.81 1.00 (0.13–3.76)	3.77 ± 2.17 3.27 (0.60-10.60)	6.50 ± 2.92 6.17 (1.23–13.67)
Height SDS	−2.11 ± 1.50 * −2.16 (−5.45-2.15)	−1.04 ± 1.37 * −1.3 (−3.87–2.39)	−0.6 ± 1.36 * −0.59 (−3.61–2.84)	−0.40 ± 1.46 * −0.64 (−3.88–3.55)
GV (cm/year)	NA	10.41 ± 3.23 * 9.94 (3.83–26.07)	7.49 ± 2.42 * 7.41 (0.73–13.48)	5.79 ± 2.71 * 5.78 (0.41–3.55)
BMI SDS	0.41 ± 1.55 0.57 (−3.92–4.24)	0.42 ± 1.38 0.44 (−2.99–4.10)	0.6 ± 1.29 * 0.80 (−2.56–4.28)	0.88 ± 1.30 * 1.12 (−2.60–3.18)
Number of patients	*n* = 142	*n* = 140	*n* = 119	*n* = 92
Age of patients at IGF1 measurement (years)	4.50 ± 3.76 2.98 (0.58–17.43)	5.85 ± 3.88 4.18 (0.85–18.74)	8.56 ± 4.21 8.17 (1.84–19.10)	11.32 ± 4.45 11.36 (3.3–19.88)
Time of rhGH therapy at IGF1 measurement (years)	NA	1.27 ± 1.00 1.00 (0.17–4.90)	3.76 ± 2.26 3.21 (0.79–9.84)	6.43 ± 2.58 6.12 (2.08–12.73)
IGF1 SDS	−0.89 ± 0.43 * −0.92 (−2.06–0.78)	1.07 ± 1.58 * 0.79 (−1.33–6.8)	1.69 ± 1.85 * 1.37 (−1.89–7.66)	1.32 ± 1.59 * 1.13 (−1.86–6.36)

PWS—Prader–Willi syndrome; rhGH—recombinant human growth hormone; *n*—number of patients; GV—growth velocity; BMI—body mass index; IGF1—insulin-like growth factor 1; NA—not applicable. * *p* < 0.05 rhGH1 vs. baseline, rhGH2 vs. rhGH1 and rhGH3 vs. rhGH2.

**Table 3:** Data during rhGH treatment in separate groups of patients with PWS, expressed as the mean value ± standard deviation scores (SDS).

rhGH Therapy	Baseline	rhGH1	rhGH2	rhGH3
**Age of patients (years) (*n*, %) ^a^**				
Group 1	1.27 ± 0.40 (*n* = 45)	2.52 ± 0.91 (*n* = 44, 98)	4.13 ± 1.55 (*n* = 35, 78)	5.70 ± 2.02 (*n* = 22, 49)
Group 2	2.93± 0.66 (*n* = 49)	4.08 ± 1.11 (*n* = 46, 94)	6.94 ± 2.56 (*n* = 45, 92)	9.89 ± 3.08 (*n* = 41, 84)
Group 3	5.91 ± 0.98 (*n* = 21)	7.44 ± 1.33 (*n* = 21, 100)	10.92 ± 2.61 (*n* =19, 91)	14.54 ± 2.96 (*n* = 15, 71)
Group 4	10.54 ± 2.44 (*n* = 32)	11.97 ± 2.7 (*n* = 29, 91)	13.94 ± 2.02 (*n* = 27, 84)	16.47 ± 2.23 (*n* = 21, 66)
**Time of rhGH therapy (years)**				
Group 1	NA	1.24 ± 0.89	2.87 ± 1.6	4.32 ± 1.97 *
Group 2	NA	1.10 ± 0.76	3.98±2.35	6.85 ± 2.84 *^,^**
Group 3	NA	1.44 ± 0.89	4.93 ± 2.26	8.57 ± 2.8 *^,^**^,^***
Group 4	NA	1.19 ± 0.73	3.77 ± 2.08	6.62 ± 2.72 *^,^***
**Height SDS**				
Group 1	−2.01 ± 1.70	−0.85±1.35	−0.65 ± 1.37	−0.42 ± 1.40
Group 2	−2.42 ± 1.23	−1.26±1.34	−0.54 ± 1.41	−0.06 ± 1.53 **
Group 3	−1.96 ± 1.37	−0.74±1.48	−0.12 ± 1.04 ***	-0.54 ± 1.27
Group 4	−1.88 ± 1.64	−1.21 ± 1.34	−0.98 ± 1.41 ***	-0.92 ± 1.41 **
**GV (cm/year)**				
Group 1	NA	12.08 ± 3.66 *	8.72 ± 2.19 *	7.77 ± 2.06 *
Group 2	NA	10.45 ± 3.02 *^,^**	8.17 ± 2.10 **	6.28±2.24 *^,^**
Group 3	NA	9.43 ± 1.34 *	6.81 ± 1.3 *^,^**^,^***	4.89 ± 2.96 *
Group 4	NA	8.51 ± 2.59 *^,^**	5.22 ± 2.23 *^,^**^,^***	3.29 ± 1.91 *^,^**
**BMI SDS**				
Group 1	−0.62 ± 1.82 *	−0.20 ± 1.59 *	0.04 ± 1.56 *	0.16 ± 1.28 *
Group 2	0.46 ± 1.33 *^,^**	0.55 ± 1.38 *	0.75 ± 1.22 *	0.95 ± 1.37 *
Group 3	1.03 ± 1.10 *	0.80 ± 0.97 *	0.93 ± 1.19 *	0.93 ± 1.35 *
Group 4	1.36 ± 0.54 *^,^**	0.8 ± 0.89 *	1.12 ± 0.81 *	1.45 ± 0.74 *
**Age of patients at IGF1 measurement (years) (n) ^a^**				
Group 1	1.27 ± 0.41 (*n* = 44)	2.48 ± 0.94 (*n* = 41)	3.99 ± 1.38 (*n* = 29)	6.21 ± 1.69 (*n* = 19)
Group 2	2.92 ± 0.67 (*n* = 47)	4.31 ± 1.61 (*n* = 48)	7.48 ± 2.77 (*n* = 44)	9.56 ± 2.74 (*n* = 35)
Group 3	5.83 ± 0.93 (*n* = 20)	7.20 ± 0.95 (*n* = 20)	10.77 ± 2.92 (*n* = 19)	14.28 ± 2.51 (*n* = 15)
Group 4	10.61 ± 2.45 (*n* = 31)	11.82 ± 2.56 (n=31)	13.47 ± 2.16 (*n* = 29)	16.29 ± 2.12 (*n* = 23)
**Time of rhGH therapy at IGF1 measurement (years)**				
Group 1	NA	1.20 ± 0.91	2.72 ± 1.37 *	4.87 ± 1.56 *
Group 2	NA	1.33 ± 1.28	4.44 ± 2.48 *^,^**	6.61 ± 2.61 *^,^**
Group 3	NA	1.28 ± 0.70	4.70 ± 2.5 *^,^***	8.27 ± 2.54 *^,^**^,^***
Group 4	NA	1.27 ± 0.84	3.02 ± 1.99 **^,^***	6.24 ± 2.54 ***
**IGF1 SDS**				
Group 1	−0.85 ± 0.24 *	0.43±1.32 *	1.27 ± 1.41 *	1.74±1.82
Group 2	−0.81 ± 0.35 **	1.28 ± 1.61 *	2.12±1.93 *^,^**	1.64 ± 1.63 **
Group 3	−0.86 ± 0.58	1.36 ± 1.22 *	2.82 ± 2.10 *^,^***	0.94 ± 1.33
Group 4	−1.08 ± 0.58 *^,^**	1.40 ± 1.86 *	0.81 ± 1.43 **^,^***	0.73 ± 1.32 **

PWS—Prader–Willi syndrome; rhGH—recombinant human growth hormone; *n*—number of patients; GV—growth velocity; BMI—body mass index; IGF1—insulin-like growth factor 1; NA—not applicable; *p* < 0.05*:* ^a^ age of diagnosis and age of IGF1 measurement between all groups, except group 3 vs. 4 for age of IGF1 measurement at rhGH3; * group 1 vs. 2, 1 vs. 3 and 1 vs. 4; ** group 2 vs. 3 and 2 vs. 4; *** group 3 vs. 4.

**Table 4:** Metabolic assessment: insulin and glucose in the OGTT, HOMA-IR and lipid profile (mean value ± standard deviation score (SDS)).

Results	Whole Cohort	Group 1	Group 2	Group 3	Group 4
Number of patients	*n* = 91	*n* = 18	*n* = 33	*n* = 18	*n* = 22
Age of patients (years) ^a^	9.09 ± 4.46	3.62 ± 1.40	7.55 ± 2.72	11.00 ± 2.8	14.69 ± 2.04
Time of rhGH therapy (years)	4.13 ± 2.60	2.32 ± 1.30 *	4.44 ± 2.45 *	4.95 ± 3.00 *	4.34 ± 2.79 *
Glucose 0 min (mg/dL)	78.75 ± 10.72	74.61 ± 11.94 *	75.54 ± 10.43 **	84.00 ± 7.90 *^,^**	82.95 ± 9.33 *^,^**
Glucose 120 min (mg/dL)	112.04 ± 25.02	100.44 ± 20.2 *	120.39 ± 25.58 *	108.67 ± 27.33	111.77 ± 22.65
Insulin 0 min (uIU/mL)	10.06 ± 7.28	5.39 ± 3.64 *	9.66 ± 8.64 *^,^**	11.14 ± 4.33 *	13.23 ± 7.46 *^,^**
Insulin 120 min (uIU/mL)	41.65 ± 33.13	18.60 ± 14.07 *	46.79 ± 41.04 *	49.77 ± 27.29 *	46.55 ± 28.94 *
HOMA IR	2.07 ± 1.71	1.02 ± 0.74 *	1.92 ± 1.97 **	2.30 ± 0.90 *^,^**	2.91 ± 1.90 *^,^**
TC (mg/dL)	184.3 ± 33.23	191.16 ± 39.40	177.87 ± 31.22	186.76 ± 28.83	186.28 ± 33.93
HDL-C (mg/dL)	56.36 ± 12.64	53.78±11.26	56.80 ± 12.75	55.26 ± 10.81	59.00 ± 15.23
LDL-C (mg/dL)	111.60 ± 32.28	120.81 ± 32.05	106.36 ± 33.69	115.14 ± 28.33	109.22 ± 33.70
TG (mg/dL)	89.85 ± 39.24	83.97 ± 32.92	84.66 ± 39.08	97.83 ± 38.90	98.98 ± 46.16

OGTT—oral glucose tolerance test; HOMA-IR—Homeostasis Model Assessment of Insulin Resistance; *n*—number of patients; rhGH—recombinant human growth hormone; TC—total cholesterol; HDL-C—high-density lipoprotein cholesterol; LDL-C—low-density lipoprotein cholesterol; TG—triglycerides; *p* < 0.05: ^a^ age of patients between all groups; * group 1 vs. 2, 1 vs. 3 and 1 vs. 4; ** group 2 vs. 3 and 2 vs. 4.

**Table 5:** Evaluation of BA/CA (mean value ± standard deviation score (SDS)) in the whole group and separate groups.

Results	Whole Cohort	Group 1	Group 2	Group 3	Group 4
Number of patients	*n* = 89	*n* = 23	*n* = 29	*n* = 16	*n* = 21
Age of patients (years) ^a^	8.58 ± 4.79	3.32 ± 1.37	7.22 ± 2.86	10.91 ± 2.68	14.44 ± 2.66
Time of rhGH therapy (years)	3.69 ± 2.61	2.02 ± 1.29 *	4.29 ± 2.67 *	4.81 ± 2.70 *	3.85 ± 2.83 *
BA/CA	0.90 ± 0.22	0.66 ± 0.16 *	0.96 ± 0.21 *	1.05 ± 0.14 *	1.00 ± 0.10 *

BA/CA—bone age/chronological age index; *n*—number of patients; rhGH—recombinant human growth hormone; *p <* 0.05: ^a^ age of patients between all groups; * group 1 vs. 2, 1 vs. 3 and 1 vs. 4.

## Data Availability

The data presented in this study are available on request from the corresponding author. The data are not publicly available due to ethical restrictions.
